# Of the article: Microbiological assessment of a herbal mouth rinse for treating chronic generalized periodontitis

**DOI:** 10.1038/s41598-025-22028-9

**Published:** 2025-10-31

**Authors:** Svetlana Razumova, Sergey Panov, Anna Marakhova, Irina Koroleva, Ekaterina Mikhailova, Anzhela Brago, Haydar Barakat

**Affiliations:** 1 Department of Propedeutics of Dental Diseases, Medical Institute, Peoples’ Friendship University of Russia named after Patrice Lumumba (RUDN University), 6 Miklukho-Maklaya Street, Moscow, 117198 Russian Federation; 2 Institute of Biochemical Technology and Nanotechnology, Peoples’ Friendship University of Russia named after Patrice Lumumba (RUDN University), 6 Miklukho-Maklaya Street, Moscow, 117198 Russian Federation; 3 Department of Molecular Microbiology, Institute of Experimental Medicine, Saint Petersburg, acad. Pavlov Street, 12, Saint Petersburg, 197022 Russian Federation; 4 Department of Conservative Dentistry, Saint Petersburg State University, Universitetskaya Embankment, 7-9, Saint Petersburg, 199034 Russian Federation

**Keywords:** Chronic generalized periodontitis, Herbal mouthwash, Chlorhexidine, Periodontal pathogens, PCR diagnostic, Dentistry, Oral microbiology, Periodontics

## Abstract

Periodontal diseases remain highly prevalent across age groups, often aggravated by poor oral hygiene, occlusal anomalies, and defective restorations. This study evaluated the effectiveness of Art-Dentale Expert mouthwash against major periodontopathogens in patients with mild chronic generalized periodontitis (CGP) using PCR diagnostics. Thirty-nine patients with CGP were randomized into a main group (Art-Dentale Expert) and a control group (Chlorhexidine). Gingival pocket samples were collected before treatment and at 7 days, 1 month, and 6 months. Four periodontopathogens (P. gingivalis, T. forsythia, T. denticola, and P. intermedia) were analyzed. The results obtained were processed statistically. Both rinses significantly reduced P. gingivalis and T. forsythia at 7 days, with Chlorhexidine showing a sharper initial decline in P. gingivalis. At 6 months, P. intermedia was completely absent in the Art-Dentale Expert group (*p* = 0.0209), whereas it persisted in 10% of controls. Art-Dentale Expert demonstrated sustained efficacy, particularly against T. denticola and P. intermedia. These findings support its role as an effective adjunct in periodontal therapy.

## Introduction

Periodontitis is a chronic inflammatory disease that affects the supporting structures of the teeth and is a leading cause of tooth loss in adults worldwide. Beyond its oral impact, it has been associated with systemic conditions such as diabetes mellitus, cardiovascular disease, and adverse pregnancy outcomes. The disease is initiated and sustained by a complex interaction between microbial biofilms and the host immune-inflammatory response^1^.

Epidemiological studies confirm a high prevalence of periodontal disease across all age groups, often linked to poor oral hygiene, dental anomalies, restorative complications, long-term medication use, and reduced immunity^2–5^. Microbiological studies have identified a wide variety of microorganisms in dental plaque, with pathogenicity strongly associated with gram-negative anaerobic species. In particular, Porphyromonas gingivalis, Tannerella forsythia, and Treponema denticola (“red complex”), as well as Prevotella intermedia and others from the “orange complex,” are considered the most virulent periodontopathogens^6–10^.

Accurate identification of these pathogens has become possible with polymerase chain reaction (PCR) diagnostics, which enables precise detection and monitoring of microbial shifts during therapy^11^. Traditional approaches to suppressing these pathogens include chemical antiseptics such as chlorhexidine, but their long-term use may be associated with adverse effects (tooth staining, altered taste, mucosal irritation). Consequently, there is increasing interest in herbal alternatives that combine antimicrobial, anti-inflammatory, analgesic, and wound-healing properties^12–16^.

Despite these potential benefits, the evidence base for herbal mouth rinses in periodontitis remains limited, and few studies have directly compared them with standard agents^17–19^. To address this gap, we developed a novel herbal formulation, Art-Dentale Expert mouth rinse (Patent), containing extracts of eucalyptus leaves, thyme herb, and pomegranate pericarp in equal parts, combined with a water–propylene glycol blend and standard stabilizing agents^20,21^.

The aim of this study was to evaluate the effectiveness of Art-Dentale Expert mouth rinse on major periodontopathogens in patients with mild chronic generalized periodontitis (CGP), using PCR diagnostics.

## Materials and methods

The ethics committee at the Medical Institute of Peoples’ Friendship University of Russia approved this study (Protocol 06 on 31.10.2019). This experimental study was performed to evaluate the “Art-Dentale Expert” mouth rinse in the treatment of chronic generalized periodontitis. The mouth rinse was manufactured by “KorolevPharm” LLC (Russia) and contains extracts of thyme herb, pomegranate pericarp, and eucalyptus leaves.

The study protocol was registered with thaiclinicaltrials.org on 04 May 2025 with the registration number: TCTR20250504001.

### Study design and participants

This study included 40 patients (23 women and 17 men) aged 18 to 44 years (mean age 36.5 ± 7.02) with mild CGP (K 05.31). The sample size (*n* = 40; 20 per group) was determined a priori using power analysis to ensure adequate statistical sensitivity. Assuming a large effect size (Cohen’s d = 0.8), a significance level of 0.05, and power of 80%, a minimum of 40 participants was deemed sufficient. This number also aligned with clinical feasibility and available resources. One patient did not respond to follow-up, resulting in a final count of 39 patients (23 women and 16 men).

The study was conducted in accordance with the Declaration of Helsinki (1975, revised in 2013), and was approved by the ethics committee of Peoples’ Friendship University of Russia. It was performed at the dental clinics of Peoples’ Friendship University of Russia and the laboratories of Saint Petersburg State University. Informed consent was obtained from all patients.

Patients were assigned to two groups: a main group and a control group (Table 1).

**Table 1:** Distribution of patients in the main and control groups by gender and age.

Group	Gender	Number of patients (%)	Main age
Main (“Art-Dentale Expert” mouth rinse)	F	11 (57.9%)	36,3 ± 0,97
	M	8 (42.1%)	
Control (0.05% chlorhexidine bigluconate solution)	F	12 (60%)	36,9 ± 1,41
	M	8 (40%)	

The main group included 19 patients with mild CGP, whose treatment included local use of the “Art-Dentale Expert” mouth rinse. The control group consisted of 20 patients with mild CGP, whose treatment included the use of the 0.05% chlorhexidine bigluconate solution. All patients used the rinse twice daily for 1 min after brushing their teeth for 7 days.

Patients in both groups were comparable in age, gender, and concomitant somatic pathology. The diagnosis of periodontal diseases was carried out in accordance with the International Classification of Diseases (ICD-10). All treatment measures were conducted based on medical indications.

### Inclusion and exclusion criteria

Inclusion criteria were a reliable diagnosis of mild CGP and informed voluntary consent from the patient. Exclusion criteria included the presence of bad habits (smoking); the presence of orthodontic appliances; severe concomitant pathology of internal organs with functional insufficiency; diabetes mellitus; malignant or benign neoplasms of any localization; HIV infection; active tuberculosis; and the patient’s refusal to be examined.

### Clinical procedures

All patients underwent clinical and radiological examinations, which included collecting medical history, assessment of dental and oral health, and recording periodontal parameters before and after treatment, such as plaque index, gingival index, bleeding on probing, probing depth, and clinical attachment level. In terms of clinical manifestations of the disease, the patients were comparable.

Patients were treated according to a standardized regimen: patient education on oral hygiene, teeth cleaning according to the Baas technique, and optimal selection of care products, followed by subsequent control. To standardize individual oral hygiene, all patients were recommended to use Biorepair Total Protection toothpaste (Biorepair, Bologna, Italy), Biorepair CURVE Total Protection toothbrush (Protezione Totale), and interdental brushes.

After individual oral hygiene education, professional hygiene was conducted according to the Guided Biofilm Therapy (GBT) protocol. Scaling and root planing were performed, and as indicated, selective grinding of teeth, correction of overhanging edges of fillings, and polishing were performed. Subsequently, patients in the main group were prescribed the “Art-Dentale Expert” mouth rinse, while patients in the control group were prescribed the 0.05% chlorhexidine bigluconate solution for 7 days. Treatment was conducted by researcher (S.P.).

### Microbiological assessment

PCR diagnostics were performed at the Federal State Budgetary Educational Institution of Higher Education “Saint Petersburg State University” after 7 days, 1 month, and 6 months.

On the day of sample collection, patients were instructed not to brush their teeth in the morning and to avoid using any medicated rinses. Microbiological samples were collected from the gingival pockets of patients in both groups using Absorbent Paper Points (Euronda, Italy) (size No. 25) sterile paper endodontic absorbents, which were inserted into the patient’s gingival pockets for 10–15 s. The obtained material was placed in a sterile, sealed Eppendorf tube and stored at −20 °C. The samples were obtained from the gingival pockets of the mandibular anterior group of teeth by one specialist (S.P.) following a standardized schema after 7 days, 1 month, and 6 months, with preliminary drying of the tooth surfaces in the morning on an empty stomach without prior brushing.

To isolate DNA from biological material, the Express-DNA-Bio kit (Alkor Bio, Russia) was used according to the protocol provided. Primers designed and tested in previous studies were utilized^21^. PCR was performed using the Tertsik amplifier (DNA-Technologies, Russia). The device was programmed for active temperature control in the solution: denaturation cycle at 94 °C for 15 s, primer annealing cycle at 94 °C for 15 s, DNA elongation cycle at 72 °C for 25 s. This cycle was repeated 35 times, followed by an incubation at 72 °C for 5 min.

DNA electrophoresis was conducted in a 1% agarose gel using a horizontal apparatus “WideMini-SubCell GT Cell-170–4468” (BioRad, USA) with Tris buffer (ThermoScientific, Germany). The electrophoresis lasted 40 min, and the voltage was set to 120 V with a gel area of 150 cm². To visualize DNA, ethidium bromide solution (0.5 µg/ml) was added to the gel. Visualization of the electrophoresis results was performed under ultraviolet light using the “iBright FL1500” (ThermoFisher, USA) visualization system. To calculate the molecular weights of the studied DNA fragments, the DNA marker “100 bp Plus DNA ladder” was utilized.

### Statistical analysis

Data were analyzed using STATISTICA software. Nonparametric tests were applied due to sample size and distribution characteristics. Between-group comparisons were performed using the Mann–Whitney U test; within-group changes were analyzed with the Wilcoxon signed-rank test. To account for multiple comparisons across time points, Bonferroni correction was applied (adjusted α = 0.017). Statistical significance was set at *p* < 0.05.

## Results

PCR diagnostics of the main periodontal pathogens in patients with mild CGP in the main and control groups before treatment showed a presence of all four studied microorganisms with the dominance of P. gingivalis (Table 2).

**Table 2:** Detection of microorganisms before treatment in both groups.

Microorganism	Main group	Control group
*P. gingivalis*	94,7%	100%
*T. forsythia*	47,4%	70%
*T. denticola*	42,1%	10%
*P. intermedia*	21%	20%

### Dynamics of individual pathogens

**Fig. 1:**
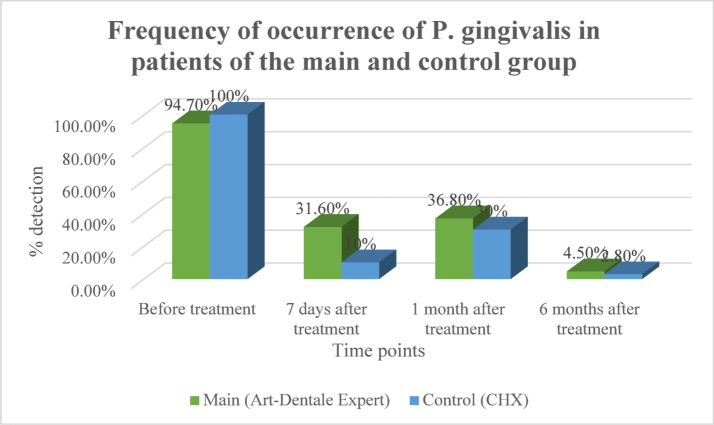
Dynamics of detection of P. gingivalis in periodontal pockets of patients in the main and control groups.

**P. gingivalis**: Both rinses significantly reduced detection after 7 days, with a sharper decline in the control group (CHX). However, by 1 month, an increase was observed in both groups, followed by a substantial decrease at 6 months (− 61.1% in the main group, − 70.0% in the control). All changes from baseline were statistically significant (*p* < 0.05) (Fig. 1).

**Fig. 2:**
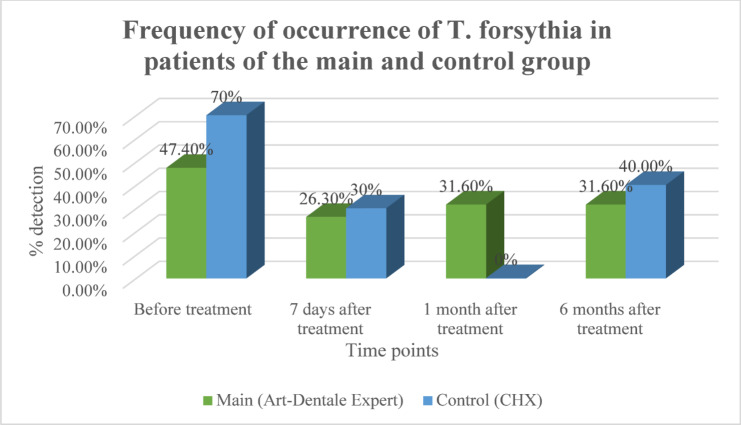
Dynamics of detection of T. forsythia in gingival pockets of patients in the main and control groups.

T. forsythia: In the main group, prevalence declined modestly and remained stable through 6 months (31.6%). In contrast, the control group showed complete elimination at 1 month, followed by a rebound to 40% at 6 months. Overall, both groups showed significant reductions compared with baseline (*p* < 0.05), with a more stable effect in the main group (Fig. 2).

**Fig. 3:**
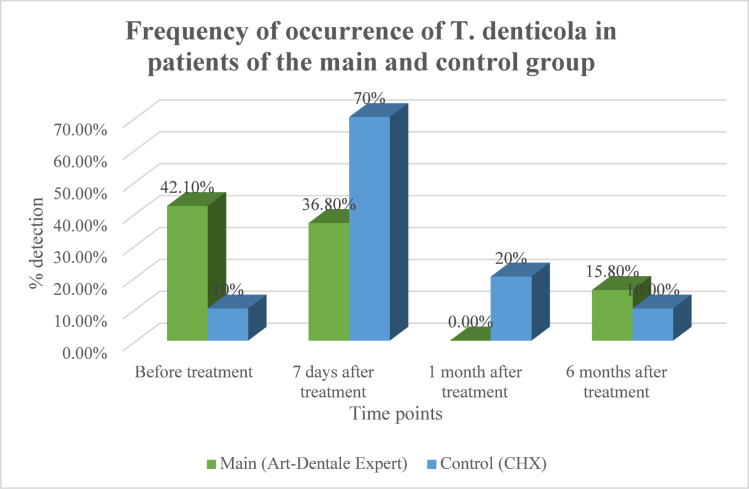
Dynamics of detection of T. denticola in gingival pockets of patients in the main and control groups.

T. denticola: The main group demonstrated a progressive decline, reaching complete elimination at 1 month (*p* = 0.0039) and remaining significantly reduced at 6 months (− 26.3% vs. baseline). By contrast, the control group showed an early increase at 7 days (*p* = 0.0003), partial reduction at 1 month, but relapse to baseline prevalence at 6 months (Fig. 3).

**Fig. 4:**
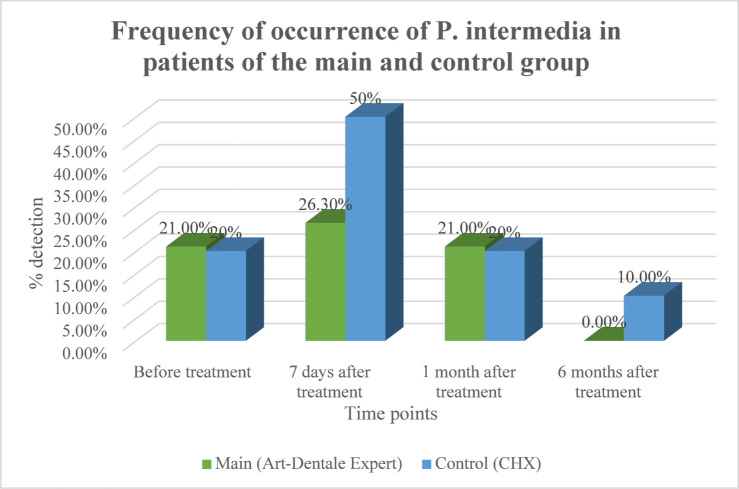
Dynamics of detection of P. intermedia in gingival pockets of patients in the main and control groups.

P. intermedia: The control group exhibited a transient increase at 7 days (20% → 50%, *p* = 0.02) before returning to baseline at 1 month. In the main group, prevalence remained low and by 6 months P. intermedia was completely absent (*p* = 0.0209), whereas 10% of control patients still tested positive (Fig. 4).

Thus, the results of PCR diagnostics demonstrate the positive effect of “Art-Dentale Expert” mouth rinse in relation to specific periodontopathogens T. denticola and P. intermedia, which is expressed in a more intensive elimination of pathogens from the gingival pockets of patients compared to patients in the control group. The dynamics of detection of periodontopathogen complexes in the gingival pockets of patients in the main and control groups after the treatment is shown in Table 3.

**Table 3:** Detection of periodontopathogen complexes (P. gingivalis, T. forsythia, T. denticola, P. intermedia) in gingival pockets.

Group	Time	Detection of periodontopathogen complexes (*P. gingivalis*,* T. forsythia*,* T. denticola*,* P. intermedia*)	Detection 1 pathogen	Detection 2 pathogens	Detection 3 pathogens	Detection 4 pathogens
		0 detection				
Main	Before treatment	5%	26%	42%	11%	16%
	After 7 days	43%	21%	5%	26%	5%
	After 1 months	16%	63%	21%	0	0
	After 6 months	53%	21%	16%	11%	0
Control	Before treatment	0	20%	60%	20%	0
	After 7 days	30%	10%	30%	30%	0
	After 1 months	40%	40%	0	20%	0
	After 6 months	50%	30%	10%	10%	0

When assessing the effectiveness of the proposed therapy, attention was paid to reducing the complexes of periodontopathogens, especially their absence and detection of a single periodontopathogen. A Single periodontopathogen was found in gingival pockets and in patients without inflammatory symptoms of periodontal tissues. The proposed treatment has led to a sharper, statistically significant (*p* < 0.05), decrease in the number of periodontopathogens in the main group after 7 days: up to 21% of cases of single periodontopathogens and up to 42% complete elimination of periodontopathogens, compared to the control group (10% of single periodontopathogens and 30% absence of periodontopathogens). This result indicates an obvious effect of using “Art-Dentale Expert” mouth rinse during complex treatment. One month after treatment, a further decrease in the number of periodontopathogens was noted in patients of the main group (Table 3). After six months, one, two and three periodontal pathogens were detected in both groups, while the periodontal pathogens were not detected in the range of 50% in both groups.

## Discussion

Herbal mouth rinses are quite effective for continuous oral care, akin to non-herbal mouth rinses based on chlorhexidine, which remains the “gold standard” in the treatment and prevention of oral diseases^23^. However, products containing chlorhexidine in various concentrations are not recommended for continuous use due to undesirable side effects, such as tooth staining and taste changes^23^. In contrast, natural ingredient-based products can be used for extended periods in daily oral care. The active ingredients in herbal products can penetrate biofilms and prevent plaque accumulation, thereby minimizing bacterial colonization on tooth surfaces^24^. They exhibit antimicrobial efficacy against dental caries and periodontal pathogens while reducing the development of resistance to medicinal products due to their synergistic combinations^25^. Additionally, herbal extracts can inhibit osteoclast differentiation and the expression of pro-inflammatory cytokines^24^, thereby suppressing bone resorption in periodontitis. Herbal mouthwashes may also decrease the adhesion of microbes to sutures following periodontal surgery, potentially improving healing^26^.

The present study demonstrated that Art-Dentale Expert, a mouth rinse containing eucalyptus, thyme, and pomegranate extracts, significantly reduced the detection of key periodontopathogens, with sustained suppression of T. denticola and complete elimination of P. intermedia at six months. These findings support the potential of herbal rinses as effective adjuncts in periodontal therapy. The antimicrobial activity of Art-Dentale Expert likely arises from the synergistic action of its components. Eucalyptus contains eucalyptol and flavonoids with antibacterial and anti-inflammatory properties, reducing bacterial adhesion and inhibiting cytokine release. Thyme provides thymol, which disrupts microbial membranes and biofilms, enhancing plaque control. Pomegranate is rich in polyphenols and tannins, which interfere with bacterial enzymes, reduce oxidative stress, and promote gingival healing. Together, these extracts can penetrate biofilms, reduce microbial colonization, and suppress host inflammatory responses, thereby creating conditions conducive to periodontal stability [^20-22^].

Numerous studies have investigated herbal oral products for their potential to reduce plaque accumulation and gingival inflammation, collectively demonstrating benefits in both clinical and microbiological outcomes. Suchetha and Bharwani evaluated Periocare^®^ Gum Massage Powder^27^, Vajrabhaya et al. assessed a herbal toothpaste containing Aloe vera^28^, and Ardakani et al. tested a herbal mouthwash formulated from Myrtus communis, Quercus brantii, Punica granatum, Portulaca oleracea, and Boswellia serrata^29^, with all reporting improvements in periodontal health. Our findings are consistent with the review by Khan et al. (2023), which concluded that herbal agents may serve as effective alternatives to conventional therapy due to their favorable safety profiles and reduced risk of drug resistance^30^. Comparisons of herbal mouth rinses with chlorhexidine against facultative anaerobes such as Enterobacter cloacae, Actinomyces viscosus, and Eikenella corrodens^31^, as well as against Streptococcus mutans, Streptococcus sanguinis, and Aggregatibacter actinomycetemcomitans^32^, indicated superior antibacterial activity for chlorhexidine, findings that differ from our results—likely due to differences in the pathogens studied. Nevertheless, our outcomes parallel those of Saquib et al. (2019), who demonstrated significant antibacterial effects of Salvadora persica (Miswak) and Cinnamomum zeylanicum (Ceylon cinnamon) extracts against P. gingivalis, T. denticola, and T. forsythia when combined with antibiotics^33^. Similarly, Karygianni et al. (2016) highlighted the antimicrobial effectiveness of various plant-derived products, including Vitis vinifera, Pinus species, Coffea canephora, Camellia sinensis, Vaccinium macrocarpon, Galla chinensis, and Caesalpinia ferrea Martius, in the treatment of dental diseases^18^, further reinforcing the role of natural agents in periodontal care.

Beyond herbal products, emerging biologically based adjuncts further support the shift away from exclusive reliance on chlorhexidine. For example, Butera et al. (2023) demonstrated the value of probiotic protocols in reducing periodontal inflammation^34^, and Cosola et al. (2022) showed improved peri-implant mucositis outcomes with hypochlorite-based formulations^35^. Together, these studies highlight a growing movement toward innovative, patient-friendly interventions, with herbal rinses representing one promising approach within this wider therapeutic landscape.

In summary, the data suggest that even six months after treatment, the use of “Art-Dentale Expert” mouth rinse resulted in superior improvements in periodontal tissue health compared to the control group. This conclusion is supported by two observations: first, there was a comparable improvement in the microbiological state of the gingival pockets for both groups; second, the primary group exhibited a reduction in the number of periodontopathogens in complexes (from four to three), a dynamic that was not observed in the control group. Thus, we conclude that “Art-Dentale Expert” mouth rinse has a statistically significant positive effect, providing prolonged benefits as a maintenance therapy during comprehensive treatment of mild chronic gingivitis.

This study provides valuable insights but should be interpreted considering its strengths and limitations. Strengths include the use of PCR diagnostics for precise identification of key periodontopathogens, a longitudinal design with multiple follow-up points (7 days, 1 month, and 6 months), and standardized clinical protocols for periodontal therapy and home care, which helped reduce confounding variables. However, the relatively small sample size limits the generalizability of the findings, and patient compliance with mouth rinse use at home was not objectively monitored. In addition, the absence of randomization may have introduced allocation bias, and only four pathogens were assessed, leaving other members of the subgingival microbiome unexamined. Future randomized controlled trials with larger populations, broader microbial profiling, and longer follow-up periods are needed to confirm and extend these results.

### Clinical relevance

For clinicians, the findings suggest that Art-Dentale Expert may be considered as a maintenance rinse during comprehensive periodontal therapy, particularly for patients requiring long-term plaque control but wishing to avoid chlorhexidine’s side effects. Its sustained suppression of T. denticola and P. intermedia is clinically meaningful, as these pathogens are strongly associated with disease progression. Integration of such herbal rinses into daily oral hygiene routines may enhance patient compliance and provide lasting antimicrobial protection.

## Conclusions

The use of the “Art-Dentale Expert” mouth rinse in the complex treatment of mild CGP has a positive effect on T. denticola and P. intermedia, leading to an accelerated decrease in the frequency of detection of periodontopathogens in the gingival pockets of patients compared to the control group and to a more significant decrease in the number of periodontopathogens studied in complexes in patients of the main group with mild CGP after just 7 days compared to the control group.

### Patent

Patent No. 2,762,978 C1 Russian Federation, IPC A01G 7/00, A01G 22/60. Method for spectral identification of the herb Mansoa alliacea (Lam.): No. 2,021,115,728: declared 01.06.2021: published 24.12.2021/A. A. Elapov, A. I. Marakhova, V. Yu. Zhilkina [et al.]; applicant Federal State Autonomous Educational Institution of Higher Education “Peoples’ Friendship University of Russia”. – EDN DVKFBV.

## Data Availability

The datasets used and analyzed during the current study are available from the corresponding author on reasonable request.
